# Altered Static and Dynamic Functional Connectivity of Habenula Associated With Suicidal Ideation in First-Episode, Drug-Naïve Patients With Major Depressive Disorder

**DOI:** 10.3389/fpsyt.2020.608197

**Published:** 2020-12-16

**Authors:** Dan Qiao, Aixia Zhang, Ning Sun, Chunxia Yang, Jianying Li, Ting Zhao, Yuchen Wang, Yifan Xu, Yujiao Wen, Kerang Zhang, Zhifen Liu

**Affiliations:** ^1^Department of Psychiatry, The First Hospital of Shanxi Medical University, Taiyuan, China; ^2^The First Clinical Medical College, Shanxi Medical University, Taiyuan, China

**Keywords:** major depressive disorder, suicidal ideation, habenula, static functional connectivity, dynamic functional connectivity

## Abstract

Investigating the neurobiological mechanism of suicidal ideation (SI) in major depressive disorder (MDD) may be beneficial to prevent the suicidal behavior. Mounting evidence showed that habenula contributed to the etiology of MDD. The habenula is a key brain region that links the forebrain to midbrain, crucial for the processing of reward and aversion. The aim of the present study was to identify whether first-episode, drug-naive MDD patients with SI displayed altered habenula neural circuitry. Forty-three and 38 drug-naïve patients with first-episode MDD with or without SI (SI+/– group) and 35 healthy control subjects (HC) underwent resting-state functional magnetic resonance imaging. The whole-brain habenula static (sFC) and dynamic functional connectivity (dFC) were calculated to identify regions showing significant difference among these three groups followed by region of interest to region of interest *post hoc* analysis. For sFC, compared with SI– and HC groups, SI+ group showed decreased sFC from habenula to the precuneus and the inferior frontal gyrus. Patients with MDD displayed increased sFC from habenula to the putamen but decreased sFC to the precentral gyrus. For dFC, SI+ group showed increased dFC from habenula to the superior temporal gyrus, the precuneus, but decreased dFC to the lingual gyrus, the postcentral gyrus, when comparing with SI– and HC groups. Patients with MDD, regardless of SI, displayed decreased dFC from the habenula to the angular gyrus. These findings provide evidence that SI in first-episode, drug-naïve patients with MDD may be related to an abnormality in habenula neural circuitry, which may provide the theoretical basis of novel treatments.

## Introduction

Major depressive disorder (MDD) is a leading cause of global disease burden and disability, which is not only associated with severe mental and physical disabilities in individuals but also carries a high risk of suicide (1, 2). Suicidal ideation (SI), as the initiation of suicide-related issues, is one of the vital risk factors for the occurrence and development of suicidal behavior (SB) (3). The prevalence of having SI is much higher than that of having suicidal planning and attempts (4, 5). Previous studies have shown that 40–70% of people with MDD experienced SI, and about 10–15% of them died as a result of suicide (6). Therefore, the underlying neural mechanism of SI in MDD patients is concerning and must be advanced.

Neurobiology mechanisms of SI are closely related to abnormal levels of monoamine metabolites in the brain (7, 8). The nuclei where monoaminergic neurons gather are located mainly in the midbrain, known as the reward circuit of the brain. Neuroimaging studies have reported associations between SI, as well as SB, and abnormal activity in the serotonergic system and the reward system, including striatum and amygdala, etc. (9, 10). Taken together, these findings suggest that brain regions that regulate reward circuit may also be in the neural circuits associated with SI and SB. One possible locus is the habenula, a part of the epithalamus located at the core of the dorsal diencephalon conduction system (11). It is an important region that links the forebrain to midbrain, receiving inputs from the prefrontal cortex and basal ganglia regions and projecting directly or indirectly to target nuclei, particularly the dopaminergic ventral tegmental area (VTA), to regulate the monoaminergic system (12, 13). Thus, it is associated with a range of behaviors related to cognitive and emotional processing, such as pain and stress responses, and is essential for reward systems and goal-directed behavior (14). Besides, the habenula was found to be activated by a no-reward-predicting target, which means that the habenula is also involved in the aversive antireward circuitry (15). The activation of the habenula leads to the suppression of motor behavior by inhibiting the release of dopamine neurons when the individual is unable to obtain a reward or predicts a negative result (16). Given this nature, as a relay station between the basal ganglia and the limbic system, the habenula is not only involved in the motivation and emotional control of behavior but also plays a key role in behavioral responses induced by expected rewards (14).

A growing body of evidence suggests that the habenula is involved in the pathophysiological mechanisms of a variety of psychiatric disorders, especially MDD (17, 18). To date, there is currently little research on the association between the habenula and suicide-related issues. A recent neuroimaging study of patients with mood disorders reported that both SB and SI was associated with abnormal connectivity of the habenula and the parahippocampal gyrus, amygdala (14). Besides MDD, changing neural connectivity of the habenula to locus coeruleus in past anorexia nervosa (pAN) with suicidality has also been discovered (19). However, these studies do not make a clear distinction between SI and SB. The neural mechanisms of SI are still understudied in the field of neuropsychiatry.

Functional magnetic resonance imaging (fMRI) based on the blood-oxygen-level-dependent (BOLD) signal is widely used to record the neural activity in the brain (20). More information about brain function can be revealed through functional connectivity (FC) in neural networks (21). Till now, most FC studies in MDD patients with SI have explored static functional connectivity (sFC), which reflect a statistical correlation of BOLD signals between different brain regions and assume that the degree of connectivity strength among regions is constant over time (10, 22). Yet, a growing body of evidence shows that communication across different regions is not static throughout the resting-state scan, but rather is dynamic due to the condition-dependent nature of neural activity (23). Temporal variations in connectivity strength, which may be especially important in SI, cannot captured by sFC. As a complementary approach, dynamic functional connectivity (dFC) can reflect the dynamic characteristics of interregional BOLD signal fluctuations over temporal scales (24). This is notable, as based on previous sFC findings of SI, understanding how connections between brain regions strengthen and weaken over time could lend insight into the neural communication behind SI in MDD, especially from the perspective of temporal stability. Furthermore, results from dFC analyses have proved superior at correctly distinguishing patients with SI from those without SI and predicting the severity of SI in comparison to sFC (25). Thus, the combination of sFC and dFC can provide comprehensive explanation of brain activity. However, there are no studies regarding dFC of the habenula in MDD patients with SI.

Taken together, the present study addressed these knowledge gaps by comparing sFC and dFC of the habenula in a sample of first-episode, drug-naive MDD patients, with and without SI, and healthy control subjects. We hypothesized that, compared to MDD patients without SI and healthy control participants, those with SI would generally exhibit weaker correlational strength and less dynamic variability in correlations from the habenula to other brain regions over time, particularly in regions involved in cognitive and emotional processing, and that such abnormalities may underline SI in MDD and could be used as features to distinguish MDD patients with SI from those without SI.

## Materials and Methods

### Subjects

The study included 116 subjects aged 18–56 years: 81 drug-naïve patients with first-episode MDD and 35 HC individuals. The drug-naive, first-episode MDD participants were recruited from the Department of Psychiatry, First Hospital of Shanxi Medical University, Taiyuan, China. The HC subjects were recruited from Taiyuan, China, using advertisement in the community. All participants were evaluated by two trained psychiatrists independently to determine the presence or absence of Axis I psychiatric diagnoses using the Structured Clinical Interview for Diagnostic and Statistical Manual of Mental Disorders, Fourth Edition (DSM-IV) Axis I Disorders (SCID).

Inclusion criteria for the patients included the following: (1) met the DSM-IV diagnostic criteria of MDD and did not meet to the criteria for any other Axis I disorder, (2) in their first episode, (3) no history of any form of antidepressant treatment, (4) no history of suicide attempt, and (5) right-handedness. HC subjects with no history of any psychiatric diagnosis and suicide attempt were included. Exclusion criteria for both patients and HC controls included the following: (1) history of head injury or any neurological disorder, (2) any serious symptom of major medical disorder, (3) history of any Axis I disorder in their first-degree relatives, and (4) any MRI contraindications.

This study was approved by the Ethics Committee of the First Hospital of Shanxi Medical University. All participants were right-handed and signed informed consent.

### Clinical Measures

Participants completed a comprehensive diagnostic evaluation within 24 h prior to the functional MRI (fMRI) scan. Suicidal ideation was assessed using the Chinese version of the Beck Scale for Suicidal Ideation (BSI-CV) (26, 27), a 19-item self-report scale that assesses thought, feeling, and plan regarding suicide. The items are rated on a 3-point scale from 0 to 2 (score range, 0–38) with higher scores indicating severe SI. Items 4 and 5 are used as a screener to assess the presence of suicidal ideation (28). Eligible patients were categorized in the suicidal ideation group (SI+) if they scored >0 on items 4 or 5. Patients were included in nonsuicidal ideation group (SI–) if they scored 0 on both items 4 and 5. Besides, only patients with a positive response on either items 4 or 5 were allowed to complete the following 14 items regarding details about SI. The final subgroups included 43 patients categorized as SI+ and 38 as SI–.

For correlations between clinically related variables and neural measures, we used the Hamilton Depression Rating Scale for Depression 24 item (HAMD-24) (29), a structured, interview-based instruments, to assess the severity of depressive symptom.

### MRI Acquisition

All structural and fMRI data were acquired on an A MAGNETOM Trio Tim 3.0T (Siemens Medical Solutions, Germany) with a 12-channel birdcage head coil located at the First Hospital of Shanxi Medical University. The participants were required to keep their eyes closed but remain awake and relaxed throughout the entire scan. Restraining foam pads and rubber earplugs were used to minimize head motion and noise interference. A 3D-FLASH sequence was used to obtain high-resolution T1-weighted structure images with the following parameters: repetition time (TR) = 2,300 ms, echo time (TE) = 2.95 ms, inversion time (TI) = 900 ms, flip angle (FA) = 9°, field of view (FOV) = 225 × 240 mm^2^, acquisition matrix = 256 × 240, slice thickness = 1.2 mm, gap = 0.6 mm, voxel size = 1 × 1 × 1 mm^3^, 160 sagittal slices, and a total of 9 min 14 s. The resting state fMRI (rs-fMRI) was performed using an echo planar imaging (EPI) sequence with the following parameters: TR = 2,000 ms, TE = 30 ms, FA = 90°, FOV = 240 × 240 mm^2^, acquisition matrix = 64 × 64, slice thickness = 3 mm, gap = 3.99 mm, 212 sagittal slices, and a total of 8 min 6 s.

### Data Preprocessing

The rs-fMRI data preprocessing was carried out using Data Processing and Analysis for Brain Imaging software (DPABI; DPABI_V4.3_200401, http://rfmri.org/dpabi) (30). The first 10 volumes that allowed the participants to adapt to scanning environments were discarded. Then, the rest of the data underwent further preprocessing, which included the following seven steps: First is the slice timing; second is the realignment of functional volumes for head motion correction. Any subject with maximum head movement exceeding 2.0 mm in displacement or more than 2.0° rotation was not included in the final analysis. To assess the frame-wise head motion confound, we compared the mean framewise displacement among the three groups. Third is the spatial normalization to the standard EPI templates from Montreal Neurological Institute (MNI) (the resampled voxel size of 3 × 3 × 3 mm^3^); fourth, spatial smoothing with a Gaussian kernel of 4 mm full width at half maximum (FWHM) to decrease noise; fifth, linear detrending; sixth, temporal band-pass filtering (0.01–0.08 Hz); and seventh, nuisance covariates regression, including Fristion 24 motion parameters, cerebrospinal fluid, and white matter signals.

### Definition of Region of Interest

The habenula was selected as the region of interest (ROI) for both sFC and dFC analysis. Each ROI was drawn as a sphere with a radius of 3 mm around a coordinate (x = −2.8, y = −24.2, z = 2.3) for the left habenula and (x = 4.8, y = −24.1, z = 2.2) for the right habenula according to a previous computational fMRI study (31).

### FC Analysis

We performed a seed-based whole-brain approach to examine sFC stemming from the bilateral habenula using DPABI. The mean BOLD time course from each seed ROI was extracted, and the Pearson's correlation coefficients with the time course of all other voxels of the brain were calculated. Then, we transformed the correlation coefficients to Z values using the Fisher's r-to-z transformation.

For dFC analysis, we performed a sliding window correlation approach (32, 33) to calculate the seed-based dFC maps of each subjects using DPABI. We chose the current sliding window analysis because of the direct nature of this approach, which will contribute to the interpretation of the study, future replication, and dissemination of results (34). To avoid the introduction of spurious fluctuations, the minimum window length should be no <1/*f*_*min*_, which is deemed the minimum frequency of the time series (35). Thus, a window length of 50 TR was considered as the appropriate parameter to optimize the balance between capturing rapidly shifting dynamic relationships and achieving reliable estimates of the correlations between regions, as suggested by a prior study (25). Hence, the time course was segmented into 50 TR Hamming windows from the scanning time in steps of 5 TR. For each sliding window, the whole brain FC maps for each ROI were than conducted between the averaged time course of all voxels in the seed and the time course of all other voxels in the whole brain. Then, Fisher's z-transformation was calculated for all FC maps, yielding a various of zFC maps for each subject. The dFC was estimated by calculating the standard deviation in zFC values through windows at each voxel.

### Statistical Analysis

The demographic and clinical characteristics of the participants were analyzed using IBM SPSS Statistics for Windows, Version 22.0. We conducted one-way analyses of variance (ANOVA) to estimate the differences among the three groups in age, years of education, and chi-square tests to detect differences in gender. For the score of HAMD-24, two-sample *t* tests were used to estimate the differences among the patient group. Statistical significance was all determined by *P* < 0.05 (two tailed).

In order to compare sFC and dFC and identify abnormalities among three groups (SI+, SI–, and HC), one-way analysis of covariance (ANCOVA) was performed with age, gender, years of education, and head motion included as covariates, using DPABI. Connectivity group differences were considered significant for voxel *P* < 0.001 with cluster *P* < 0.05 [using Gaussian random field (GRF) correction]. Significant clusters identified in this analysis were then imported as target ROI in the *post hoc* ROI to target ROI analysis. We extracted mean sFC and dFC values between the left/right habenula and significant clusters for each group and conducted *post hoc* pairwise comparisons. The results were considered significant if *P* < 0.05, corrected by Bonferroni test.

To further identify the dimensional correlations between clinical symptoms and neural patterns in MDD patients with SI, Pearson's partial correlation analyses (two tailed), controlling for age, gender, and years of education, were conducted between FC (sFC/dFC) values with significant group differences and the total score of HAMD-24, and BSI-CV (*P* < 0.05, Bonferroni correction).

### Validation Analysis

To verify our findings of dFC variability obtained from 50 TR lengths of the sliding window, we further performed validation analysis for different sliding window lengths besides 50 TR. Thus, we recalculated the main results of dFC using the other two window lengths (30 and 60 TR).

Besides, the results were reanalyzed by regressing out HAMD scores to test the difference between the SI+ and SI– groups with respect to sFC and dFC.

## Results

### Demographics and Clinical Characteristics

Table 1 shows the demographic and clinical characteristics of the subjects. There were no statistical differences among the three groups in terms of age (*F* = 0.265, *P* = 0.768), gender (χ^2^ = 0.299, *P* = 0.861), years of education (*F* = 2.341, *P* = 0.101), or head motion (*F* = 0.121, *P* = 0.886). For clinical symptoms, the total score of HAMD-24 in the SI+ group were higher than in the SI– group (*t* = 3.086, *P* = 0.003).

**Table 1:** Demographics and clinical characteristics of all participants.

**Variables**	**SI+ (*n* = 43)**	**SI– (*n* = 38)**	**HC (*n* = 35)**	***F/*χ*^**2**^/t***	**P**
Age, years (mean ± SD)	33.12 ± 11.47	34.24 ± 9.36	32.57 ± 8.75	0.265	0.768
Gender (male/female)	21/22	17/21	15/20	0.299	0.861
Education, years (mean ± SD)	11.63 ± 3.53	12.24 ± 2.97	13.17 ± 2.78	2.341	0.101
BSI-CV (mean ± SD)	17.56 ± 7.17				
HAMD-24 (mean ± SD)	26.56 ± 4.15	23.79 ± 3.89		3.086	0.003
Mean FD (mean ± SD)	0.09 ± 0.05	0.10 ± 0.05	0.10 ± 0.05	0.121	0.886

*SI+, major depressive disorder patients with suicidal ideation; SI–, major depressive disorder patients without suicidal ideation; HC, healthy controls; SD, standard deviation; BSI-CV, the Chinese version of the Beck Scale for Suicidal Ideation; HAMD-24, Hamilton Depression Rating Scale for Depression 24 item; FD, framewise displacement*.

### Group Differences in sFC

**Figure 1:**
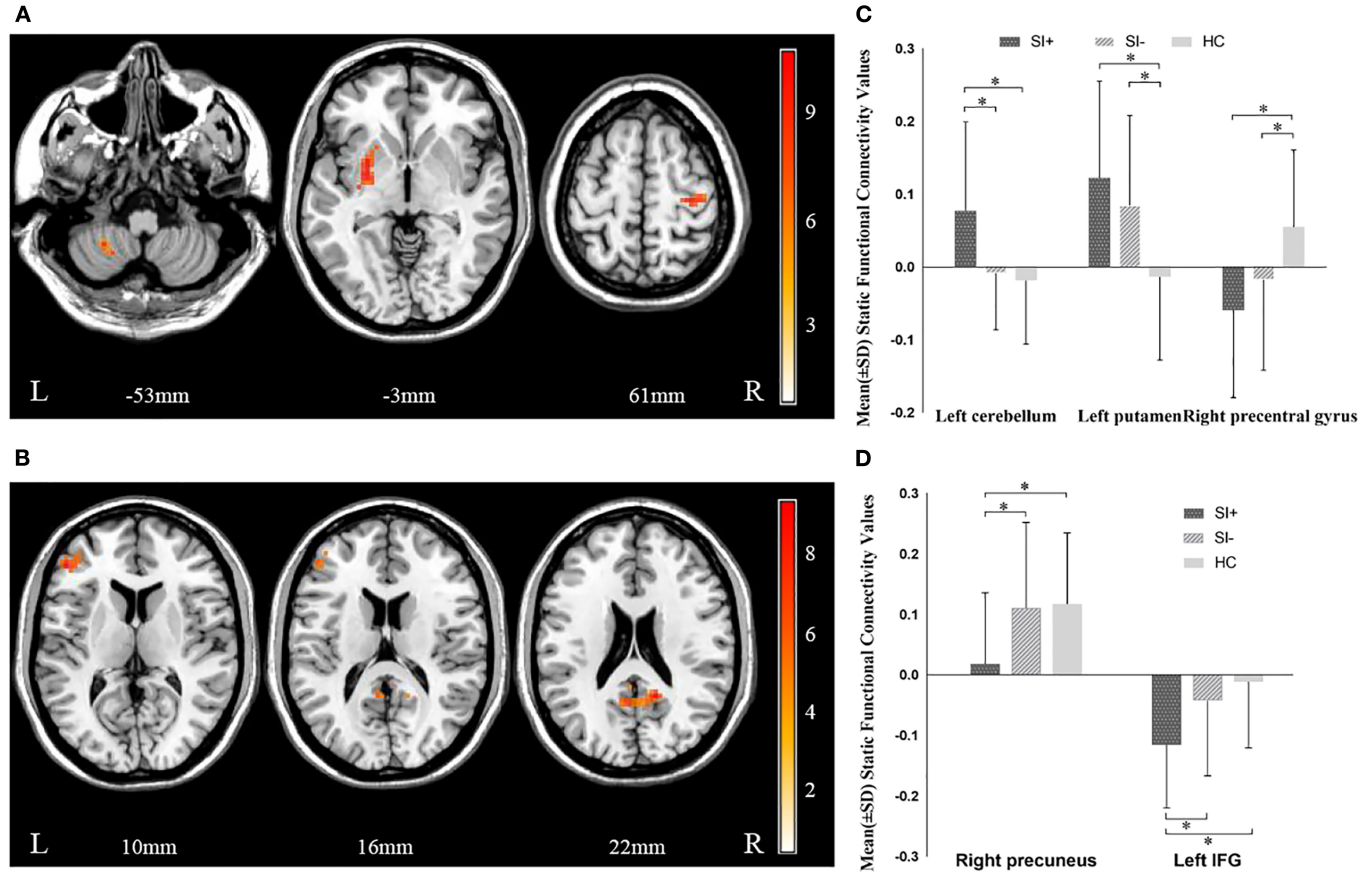
Significant differences in static functional connectivity (sFC) of **(A)** left habenula and **(B)** right habenula among the major depressive disorder with suicidal ideation (SI+), MDD without suicidal ideation (SI–), and healthy control (HC) groups. The color bars indicate the *F* value based on one-way ANOVA. Significant at *P* < 0.001, corrected by Gaussian random field (GRF) correction. *Post hoc* analysis in static functional connectivity (sFC) of **(C)** left habenula and **(D)** right habenula among the major depressive disorder with suicidal ideation (SI+), MDD without suicidal ideation (SI–), and healthy control (HC) groups. ^*^P < 0.05, corrected by Bonferroni test. IFG, inferior frontal gyrus.

Age-, gender-, years of education-, and head motion-adjusted ANCOVAs demonstrated that the three groups significantly differed in sFC between the left habenula and the left putamen (*F* = 8.280, *P* = 0.000), the left cerebellum (*F* = 10.849, *P* = 0.000), and the right precentral gyrus (*F* = 7.414, *P* = 0.000), as well as altered sFC between the right habenula and the right precuneus (*F* = 7.818, *P* = 0.000) and the left inferior frontal gyrus (IFG) (*F* = 8.149, *P* = 0.000) (Figure 1, Table 2). In *post hoc* comparisons, the SI+ group had significantly decreased sFC between the right habenula and the right precuneus (SI+ vs. SI–: *P* = 0.004; SI+ vs. HC: *P* = 0.002; SI– vs. HC: *P* = 1.000), and the left IFG (SI+ vs. SI–: *P* = 0.020; SI+ vs. HC: *P* = 0.000; SI– vs. HC: *P* = 0.749), as well as increased sFC between the left habenula and the left cerebellum (SI+ vs. SI–: *P* = 0.001; SI+ vs. HC: *P* = 0.000; SI– vs. HC: *P* = 1.000), compared with both the SI– and HC groups, but no difference was observed in sFC between the SI– and the HC groups. Compared with the HC group, both the SI+ and SI– groups showed increased connectivity between the left habenula and the left putamen (SI+ vs. SI–: *P* = 0.665, SI+ vs. HC: *P* = 0.000, SI– vs. HC: *P* = 0.022) and decreased sFC between the left habenula and the right precentral gyrus (SI+ vs. SI–: *P* = 0.438; SI+ vs. HC: *P* = 0.001; SI– vs. HC: *P* = 0.042), while no difference was observed in sFC between patient groups (Figure 1).

**Table 2:** Brain regions showing significant FC differences across the three study groups.

**Seed**	**Region**	**MNI coordinates**			**Voxels**	***F* values**
		**X**	**Y**	**Z**		
**sFC**						
Left habenula	Left cerebellum	−30	−48	−48	33	11.961
	Left putamen	−27	3	−3	48	8.063
	Right precentral gyrus	33	−21	60	36	8.370
Right habenula	Right precuneus	15	−51	21	32	10.302
	Left IFG	−48	39	9	35	9.204
**dFC**						
Left habenula	Right lingual gyrus	6	−60	−3	14	7.709
	Left precuneus	−3	−72	42	22	8.620
	Right precuneus	9	−57	60	18	10.260
Right habenula	Left STG	−60	−42	15	18	10.336
	Left angular gyrus	−48	−60	51	16	10.072
	Left postcentral gyrus	−42	−42	54	30	8.350

*MNI, Montreal Neurological Institute; FC, functional connectivity; sFC, static functional connectivity; dFC, dynamic functional connectivity; IFG, inferior frontal gyrus; STG, superior temporal gyrus*.

### Group Differences in dFC

**Figure 2:**
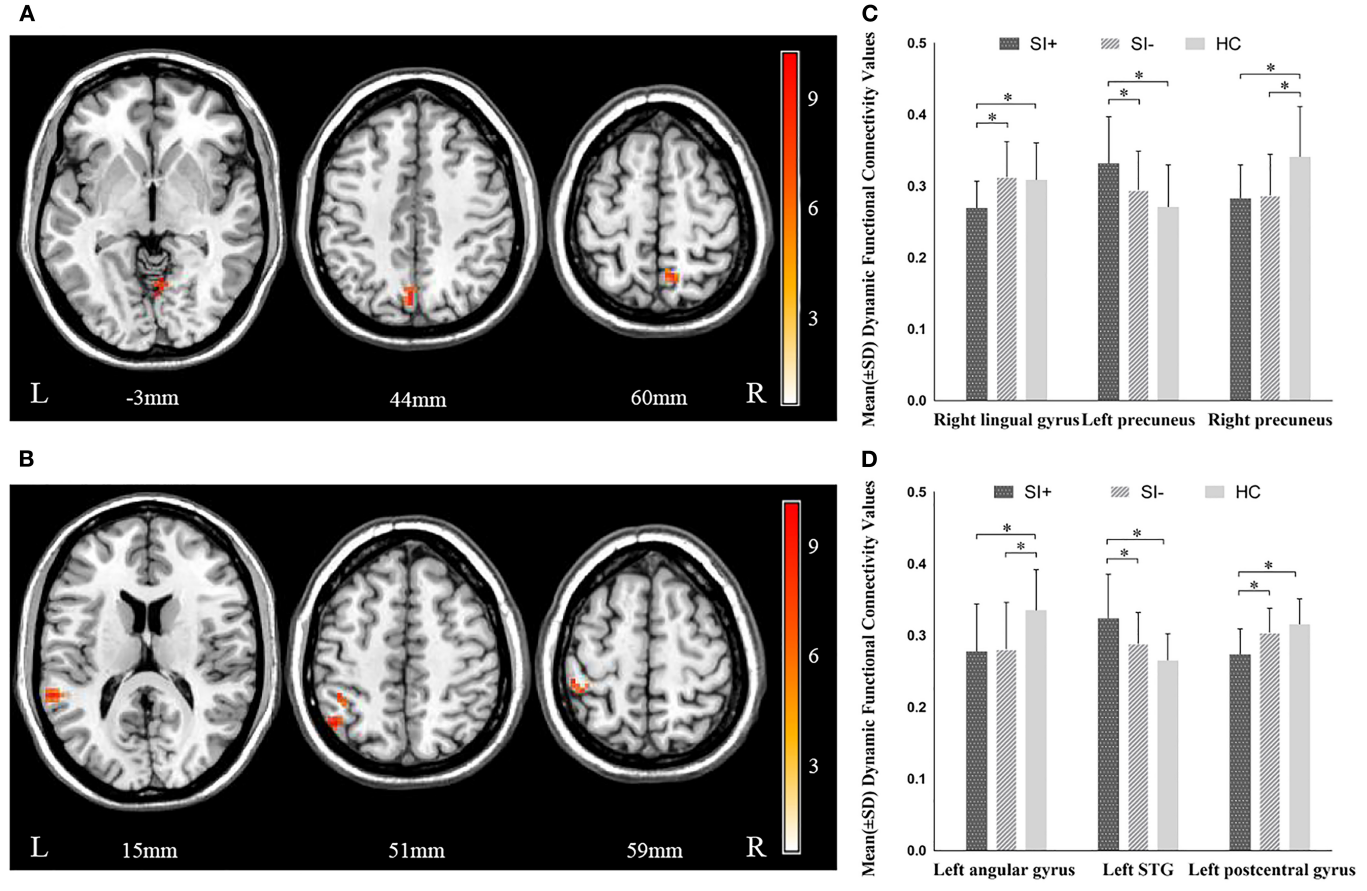
Significant differences in dynamic functional connectivity (dFC) of **(A)** left habenula and **(B)** right habenula among the major depressive disorder with suicidal ideation (SI+), MDD without suicidal ideation (SI–), and healthy control (HC) groups. The color bars indicate the *F* value based on one-way ANOVA. Significant at *P* < 0.001, corrected by Gaussian random field (GRF) correction. *Post hoc* comparison in dynamic functional connectivity (dFC) of **(C)** left habenula and **(D)** right habenula among the major depressive disorder with suicidal ideation (SI+), MDD without suicidal ideation (SI–), and healthy control (HC) groups. ^*^P < 0.05, corrected by Bonferroni test. STG, superior temporal gyrus.

Significant differences in dFC were observed among the three groups between the left habenula and the right lingual gyrus (*F* = 10.790, *P* = 0.000), the left (*F* = 10.430, *P* = 0.000), and right precuneus (*F* = 11.473, *P* = 0.000). The three groups significantly differed also in dFC between the right habenula and the left superior temporal gyrus (STG) (*F* = 14.096, *P* = 0.000), the left angular gyrus (*F* = 9.618, *P* = 0.000), and the left postcentral gyrus (*F* = 15.056, *P* = 0.000) (Figure 2, Table 2). In *post hoc* comparisons, the SI+ group exhibited significantly decreased dFC between the left habenula and the right lingual gyrus (SI+ vs. SI–: *P* = 0.000; SI+ vs. HC: *P* = 0.001; SI– vs. HC: *P* = 1.000) and between the right habenula and the left postcentral gyrus (SI+ vs. SI–: *P* = 0.001; SI+ vs. HC: *P* = 0.000; SI– vs. HC: *P* = 0.496), as well as increased dFC between the left habenula and the left precuneus (SI+ vs. SI–: *P* = 0.025; SI+ vs. HC: *P* = 0.000; SI– vs. HC: *P* = 0.244), and between the right habenula and the left STG (SI+ vs. SI–: *P* = 0.005; SI+ vs. HC: *P* = 0.000; SI– vs. HC: *P* = 0.140), compared with both the SI– and HC groups, but no difference was observed in dFC between the SI– and the HC groups. Meanwhile, compared with the HC group, both the SI+ and SI– groups showed decreased dFC between the left habenula and the right precuneus (SI+ vs. SI–: *P* = 1.000; SI+ vs. HC: *P* = 0.000; SI– vs. HC: *P* = 0.000) and between the right habenula and the left angular gyrus (SI+ vs. SI–: *P* = 1.000; SI+ vs. HC: *P* = 0.000; SI– vs. HC: *P* = 0.001), but no difference was observed in dFC between patient group (Figure 2).

### Correlation Analysis Between FC and Clinical Measurements

No significant correlations between the sFC and dFC in regions showing significant group differences, and clinical symptom measures were observed in the SI+ group (*P* > 0.05, Bonferroni correction).

### Validation Results

In this study, we used two different sliding window lengths to verify our main results of dFC. The results of the sliding window lengths of 30 and 60 TRs were similar to the results of the 50 TR we found. All validation analysis results were presented as Supplementary Material (Supplementary Tables 1, 2).

Except the left cerebellum (*P* > 0.05), the abnormalities of sFC and dFC observed in *post hoc* analysis were also found between the SI+ and SI– groups after regressing out HAMD scores (Supplementary Figure 1).

## Discussion

The present study explored neuroimaging correlates of SI in a population of first-episode, drug-naive MDD patients. To the best of our knowledge, it represents the first study to investigate differences between patients with a diagnosis of MDD, with and without SI, and healthy controls in sFC and dFC of the habenular. We found that SI+ group had significantly decreased sFC between the right habenula and the right precuneus and the left IFG. As for dFC, SI+ group had significantly decreased dFC between the left habenula and the right lingual gyrus, between the right habenula and the left postcentral gyrus, as well as increased dFC between the right habenula and the left STG and between the left habenula and the left precuneus. Moreover, patients with a diagnosis of MDD, independently of SI, exhibited altered sFC between the left habenula and the left putamen and the right precentral gyrus, as well as abnormal dFC between the left habenula and the right precuneus, and between the right habenula and the left angular gyrus.

As a key region within the reward circuit that regulates the midbrain monoamine system, the function of the habenula in humans is of great clinical relevance (31). Our data revealed that aberrant habenula functional connectivity may be involved in the pathogenesis of SI in MDD patients. The ability of individuals to maintain properly functioning reward pathways drives motivational behavior, which is critical for successful copying with challenges, while excessive sensitivity to negative stimuli promotes avoidance and isolation, thereby jeopardizing appropriate coping strategies (36). Several literatures indicate that both a “reward deficit syndrome,” clinically characterized by anhedonia and reduced motivation, along with an “enhanced anti-reward syndrome,” which noticeable as dysphoria, may be driving forces behind SI (37). Hence, due to its unique anatomical and functional location, habenula regulates reward circuits and participates in antireward circuits by directly or indirectly regulating the release of dopamine and serotonin downstream in the limbic region (14), which may be one of the neurobiological mechanisms of SI.

In our sample, sFC between the right habenula and the left IFG was decreased in MDD patients with SI. The IFG is known to be involved in response inhibition, a component of executive function, and is essential for impulse control, while SI has a clear correlation with the increase in impulsivity and neurobehavior disinhibition (38). In this context, our study suggests that the deficit in neural connections between the habenula and the IFG may play a distinct role in the etiology of SI through abnormal behavior inhibition of reward and aversion information, and more research is needed to explain this in the future.

Traditional sFC reflects average connectivity strength between regions, while dFC assesses functional connectivity variability over time. In our study, dFC provided additional information that was different from but complementary to the information for sFC. The greater the dFC, the more frequent the switching of the FC strength between the two regions. Regarding the dFC analyses, significantly decreased dFC between the habenula and the postcentral gyrus was found in first-episode MDD patients with SI compared to those without SI and normal controls. The postcentral gyrus is considered to be a region involved in somatosensory perception and representation of emotional response (39). Further, it is often associated with empathy and social perception, such as the experience of social rejection (40–42). A neuroimaging report of peer interactions demonstrated that, compared with lower SI, higher SI in depressed adolescent is linked to significantly lower activity in postcentral gyrus during peer exclusion and inclusion (43). A possible interpretation of our findings is that lower dFC (reduced switching frequency) between the habenula and somatosensory cortex may indicate an abnormal emotional response to reward stimuli, leading to feelings of rejection and lack of motivation in patients with SI. Another region we found showing decreased dFC is the lingual gyrus. Although there are few imaging evidence that the lingual gyrus may be involved in SI in MDD, smaller gray matter volumes in the left lingual gyrus have been reported in psychotic disorders with suicidality (44). The lingual gyrus, located in the visual recognition network and connected to the posterior insula, has been hypothesized to play a critical role in integrating visual information with introspective sensations or stimuli (45). This makes functional connectivity to the habenula in patients with SI possible an important feature of impairments in introspective integration processing, particularly those integration involving rewards as a main information.

In addition, we observed greater dFC between the habenula and STG in SI+ group. Although there are few studies on the role of instability of FC of STG in SI, previous studies investigating morphological and functional abnormalities of brain in patients with SI have reported altered STG connections and gray volumes (46, 47). These observations indicate that alternations in the STG may be a potential biomarker for SI in MDD. In a study of single-photon emission computed tomography (SPECT) imaging, the abnormal regional cerebral blood flow was reported in the temporal pole, which was identified as one of the regions where SI was predicted in depressed patients (48). The STG has been demonstrated to work as the primary brain area responsible for emotional processing and cognitive regulation (47, 49). Thus, we discreetly speculate that the excessive FC variability between the habenula and the STG may cause a strong response to negative stimuli and the failure to control negative emotions, resulting in the occurrence of SI.

It is also worth noting that in patients with SI, the habenula has shown remarkable lower sFC but greater dFC to the precuneus, which is closely related to fundamental cognitive functioning (50). In other words, patients with SI have “weaker” but more “flexible” connectivity between these regions. A study of fMRI showed that previous SI in MDD patients was linked to decreased brain activity in the precuneus and cuneus, during cognitive control tasks (51). The precuneus plays a vital role in a series of highly integrated tasks, especially self-processing operations (50, 52). Critically, self-processing involves distinct processes and can occur in cognition-related brain regions, especially precuneus, which is previously also implicated in one's self-awareness (53). According to the interpersonal theory of suicide (54), the coexistence of indicators of thwarted belongingness, perceived burdensomeness, and hopelessness about one's personal relationships is a proximate and sufficient cause of SI. “Thwarted belongingness” refers to feelings of alienation from a group or society, while “perceived burdensomeness” refers to the perception of oneself as a burden on others. Both these two factors involve negatively valanced, self-referential processing (52). Weaker but more flexible connectivity between habenula and precuneus in patients with SI may underlie the tendency to link reward/antireward processing with self-thoughts during the resting state.

Taken together, these findings demonstrate that MDD patients with SI exhibits abnormal FC, especially from dynamic nature. These abnormal dynamic functional connections provide additional information about the status of brain functional circuits and are sensitive measures to detect changes in brain circuits in depressed patients with SI. In addition, a better understanding of the connection between SI and brain dynamic activity can further enhance our comprehension of how temporal variations in connectivity strength promoting development of SI in patients with depression. Specifically, dynamic attributes may be a powerful neuroimaging indicator to explore the pathological changes of SI in patients with MDD and is expected to provide a new way to distinguish MDD patients with and without SI.

It is important to address several limitations in the present study. First, there was a relatively small sample size, which may affect the statistical power, but we are still to keep collecting the relevant samples for future research about SI. Second, the cross-sectional design of this study limited the ability to infer whether the altered FC of habenula was a preexisting abnormality or a consequence of SI. Longitudinal study is needed in the future to explore the direct causality of SI with related neural variables. Third, in our study, we did not collect the information about the duration and the age of first onset of patients, which also may affect the accuracy of our results. Besides, the optimal length of the sliding window for obtaining the dynamics of brain activity is still unclear, although validation analyses with different window sizes were conducted to increase consistency. Other correction models of dFC, such as the wavelet transformation coherence method (55), should be used for further analysis. Considering the high comorbidity of anxiety and depression, the exclusion of anxious individuals may reduce the generalizability of the findings. One other limitation of this study is that only MDD patients with SI were selected for this study. Given the multiple dimensions of suicide-related issues, such as suicide attempt, future efforts will be important to determine whether there are different or similar neurobiological activities between different suicide dimensions, thus providing a basis for the discovery of new and effective clinical interventions for suicide.

In conclusion, the current study presented novel results linking SI to the abnormal functional connectivity of habenula using both sFC and dFC. As such, from a neurobiological point of view, the altered sFC and dFC of the habenula in drug naive, first-episode MDD patients with SI may mediate a dysfunction in the mechanism that links the habenula with self-referential processing, response inhibition, emotional response, and introspective integration processing. Nonetheless, additional research, especially longitudinal study, focused on the neural features of different suicide dimensions is needed to explore neural biomarkers for suicide risk and to facilitate early identification and intervention.

## Data Availability Statement

The raw data supporting the conclusions of this article will be made available by the authors, without undue reservation.

## Ethics Statement

The studies involving human participants were reviewed and approved by the Ethics Committee of the First Hospital of Shanxi Medical University. The patients/participants provided their written informed consent to participate in this study.

## Author Contributions

DQ, ZL, and KZ contributed to conception and design of the study. JL, TZ, YX, and YWe collected the samples and participants' characteristics. AZ, CY, and NS organized the database. DQ and YWa analyzed data and performed the statistical analysis. DQ wrote the first draft of the manuscript. All authors contributed to manuscript revision, read, and approved the submitted version.

## Conflict of Interest

The authors declare that the research was conducted in the absence of any commercial or financial relationships that could be construed as a potential conflict of interest.
