# Supplementary material for: Altered Static and Dynamic Functional Connectivity of Habenula Associated With Suicidal Ideation in First-Episode, Drug-Naïve Patients With Major Depressive Disorder
Source: Front Psychiatry. 2020 Dec 16;11:608197. doi: 10.3389/fpsyt.2020.608197 (PMC7772142; doi:10.3389/fpsyt.2020.608197)
Supplement: Supplementary file 1 [file Table_1.DOCX]

Supplementary Material

Supplementary Table 1. Brain regions showing significant dFC differences across the three study groups in validation analysis

| Seed | Region | MNI coordinates | | | Voxels | *F* Values |
| --- | --- | --- | --- | --- | --- | --- |
|  |  | X | Y | Z |  |  |
| window lengths: 30TR | | | | | | |
| Left habenula | Right lingual gyrus | 6 | -60 | -3 | 18 | 10.025 |
|  | Left precuneus | -3 | -69 | 48 | 17 | 8.274 |
| Right habenula | Left postcentral gyrus | -42 | -42 | 54 | 31 | 9.788 |
|  | Right angular gyrus | 42 | -42 | 54 | 17 | 11.530 |
| window lengths: 60TR | | | | | | |
| Left habenula | Left precuneus | -3 | -72 | 42 | 15 | 8.375 |
|  | Right precuneus | 9 | -57 | 60 | 20 | 10.421 |
| Right habenula | Left STG | -60 | -42 | 15 | 19 | 12.428 |
|  | Left angular gyrus | -45 | -60 | 51 | 15 | 10.696 |
|  | Left postcentral gyrus | -48 | -33 | 60 | 13 | 8.708 |

Note: MNI, Montreal Neurological Institute; dFC, dynamic functional connectivity; TR, repetition time; STG, superior temporal gyrus
