# Supplementary material for: Altered Static and Dynamic Functional Connectivity of Habenula Associated With Suicidal Ideation in First-Episode, Drug-Naïve Patients With Major Depressive Disorder
Source: Front Psychiatry. 2020 Dec 16;11:608197. doi: 10.3389/fpsyt.2020.608197 (PMC7772142; doi:10.3389/fpsyt.2020.608197)
Supplement: Supplementary file 2 [file Table_2.DOCX]

Supplementary Material

Supplementary Table 2. Post-hoc analysis of dFC among three study groups in validation analysis.

| Seed | Region | *P* values | | | *F* Values |
| --- | --- | --- | --- | --- | --- |
|  |  | SI+ *vs* SI- | SI+ *vs* HC- | SI- *vs* HC |  |
| window lengths: 30TR | | | | | |
| Left habenula | Right lingual gyrus | 0.000 | 0.000 | 1.000 | 16.367 |
|  | Left precuneus | 0.034 | 0.000 | 0.620 | 8.251 |
| Right habenula | Left postcentral gyrus | 0.007 | 0.000 | 0.075 | 14.681 |
|  | Right angular gyrus | 1.000 | 0.000 | 0.000 | 12.117 |
| window lengths: 60TR | | | | | |
| Left habenula | Left precuneus | 0.011 | 0.000 | 0.441 | 10.877 |
|  | Right precuneus | 1.000 | 0.000 | 0.000 | 12.349 |
| Right habenula | Left STG | 0.001 | 0.000 | 0.151 | 16.375 |
|  | Left angular gyrus | 1.000 | 0.001 | 0.000 | 10.226 |
|  | Left postcentral gyrus | 0.024 | 0.000 | 0.663 | 8.129 |

Note: dFC, dynamic functional connectivity; SI+, major depressive disorder patients with suicidal ideation; SI-, major depressive disorder patients without suicidal ideation; HC, healthy controls; TR, repetition time; STG, superior temporal gyrus.
