# Supplementary material for: Altered Static and Dynamic Functional Connectivity of Habenula Associated With Suicidal Ideation in First-Episode, Drug-Naïve Patients With Major Depressive Disorder
Source: Front Psychiatry. 2020 Dec 16;11:608197. doi: 10.3389/fpsyt.2020.608197 (PMC7772142; doi:10.3389/fpsyt.2020.608197)
Supplement: Supplementary file 3 [file Table_3.DOCX]

Supplementary Material

Supplementary Fig 1. HAMD score regression in static(sFC) of left habenula (A) and right habenula (B) and dynamic functional connectivity(dFC) of left habenula (C) and right habenula (D). *P < 0.05. SI+, major depressive disorder patients with suicidal ideation. SI-, major depressive disorder patients without suicidal ideation. IFG, inferior frontal gyrus. STG, superior temporal gyrus.
